# Correlation between psychotic risk and depressive “cognitive” symptoms in adolescence

**DOI:** 10.1192/j.eurpsy.2022.1753

**Published:** 2022-09-01

**Authors:** A. Maffucci, S. Cesario, V. Mammarella, G. Colafrancesco, M. Ferrara, A. Raballo, E. Monducci

**Affiliations:** 1 Sapienza University of Rome, Department Of Human Neuroscience, Section Of Child And Adolescent Neuropsychiatry, Rome, Italy; 2 Università degli Studi di Perugia, Faculty Of Medicine, Perugia, Italy

**Keywords:** Self Disorders, Ultra High Risk, psychosis prevention, depressive symptoms

## Abstract

**Introduction:**

Prevention of disorders has become a central element of psychiatric research and clinic. Currently, Ultra High Risk (UHR) criteria are internationally recognized for psychiatric risk assessment. Self Disorders (SD) aroused particular interest because they were found to be specific to schizophrenic spectrum disorders and a marker of vulnerability for psychotic onset.

**Objectives:**

To evaluate the correlation between psychotic risk and depressive symptoms in at-risk adolescent population.

**Methods:**

We collected data from 80 patients, aged 14-18, with sufficient skills in the Italian language and an IQ ≥70, excluding patients with disorders related to direct effects of a general medical condition or substance. Psychodiagnostic evaluation included K-SADS-PL, SIPS/SOPS, EASE (for the assessment of SDs) and the CDSS (for the assessment of Depression).

**Results:**

35 subjects have UHR criteria, while 45 do not have a psychotic risk syndrome or psychotic features. Between the two groups there is a significant difference in the total SCORE of EASE, in domains 1, 2 and 5. In addition, a positive correlation between SDs and depressive symptoms emerged, in particular with pathological guilt and with reference ideas of guilt.

**Conclusions:**

The results confirm the validity of SDs for early detection of psychosis. Depressive features appear to be associated with the presence of abnormalities of experience. This results suggest a close care and monitoring of depressive symptoms in adolescence, because they can mask disorders of different nature, particularly pathological guilt and guilty ideas of reference that are depressive “cognitive” symptoms more correlate with psychotic risk.

**Disclosure:**

No significant relationships.

